# Stability Kinetics of Anthocyanins of Grumixama Berries (*Eugenia brasiliensis* Lam.) during Thermal and Light Treatments

**DOI:** 10.3390/foods12030565

**Published:** 2023-01-28

**Authors:** Elivaldo Nunes Modesto Junior, Mayara Galvão Martins, Gustavo Araujo Pereira, Renan Campos Chisté, Rosinelson da Silva Pena

**Affiliations:** 1 Graduate Program of Food Science and Technology (PPGCTA), Institute of Technology (ITEC), Federal University of Pará (UFPA), Belém 66075-110, PA, Brazil; 2 Faculty of Food Engineering (FEA), Institute of Technology (ITEC), Federal University of Pará (UFPA), Belém 66075-110, PA, Brazil

**Keywords:** Amazonian fruits, bioactive compounds, light stability, food processing

## Abstract

Grumixama (*Eugenia brasiliensis* Lam.) are red-colored fruits due to the presence of anthocyanins. In this paper, anthocyanin-rich extracts from grumixama were submitted to different temperatures and light irradiations, with the aim of investigating their stabilities. The thermal stability data indicated that a temperature range from 60 to 80 °C was critical to the stability of the anthocyanins of the grumixama extracts, with a temperature quotient value (Q_10_) of 2.8 and activation energy (E_a_) of 52.7 kJ/mol. The anthocyanin-rich extracts of grumixama fruits showed the highest stability during exposure to incandescent irradiation (50 W), followed by fluorescent radiation (10 W). The t_1/2_ and k were 59.6 h and 0.012 h^−1^ for incandescent light, and 45.6 h and 0.015 h^−1^ for fluorescent light. In turn, UV irradiation (25 W) quickly degraded the anthocyanins (t_1/2_ = 0.18 h and k = 3.74 h^−1^). Therefore, grumixama fruits, and their derived products, should be handled carefully to avoid high temperature (>50 °C) and UV light exposure in order to protect the anthocyanins from degradation. Furthermore, grumixama fruits showed high contents of anthocyanins that can be explored as natural dyes; for example, by food, pharmaceutical and cosmetic industries. In addition, the results of this study may contribute to the setting of processing conditions and storage conditions for grumixama-derived fruit products.

## 1. Introduction

*Eugenia brasiliensis* Lam., commonly known as grumixama and Brazilian cherry, is a tree from the Brazilian coastal forests that belongs to the genus *Eugenia*, which is one of the largest genera in the family Myrtaceae with about 350 species [1]. Grumixama fruits have red peel due to the presence of anthocyanins, especially cyanidin 3-glucoside and delfinidin 3-glucoside [2]. The grumixama fruits are commonly used to prepare homemade foods, such as frozen fruits, frozen pulps and jellies. Furthermore, anthocyanins recovered from peels of purple grumixama were reported to have great potential to be used as a natural colorant in the food industry [3].

Anthocyanins are secondary metabolites that protect plants against various biotic and abiotic stresses [4]. They are water-soluble pigments, with high antioxidant capacity, belonging to a polyphenol-based flavonoids family, due to their characteristic C_15_ skeleton based on a C_6_-C_3_-C_6_ core structure [5]. Chemically, anthocyanins are polyhydroxylated and/or polymethoxylated glycosides derived from the 2-phenylbenzopyrilium ion (flavylium cation), which gives anthocyanins their mostly red coloring, at low pH values, which distinguishes them from other types of flavonoids [6,7]. Anthocyanins are naturally found in a wide number of foods, including fruits and vegetables. They are predominantly found in berries, currants, grapes, tropical fruits, red to purplish, and blue-colored leafy vegetables, grains, roots, and tubers [8].

Anthocyanins are widely studied as bioactive compounds to manage and/or prevent the onset/development of several chronic degenerative diseases, including cardiovascular diseases, cancers, type-2 diabetes mellitus, neurodegenerative diseases, and dyslipidemias [9]. On the other hand, anthocyanins are highly unstable and susceptible to degradation during food processing and storage, which varies widely according to their chemical structure and concentration within the food matrix. These characteristics strongly influence the anthocyanin content in the final product and their benefits to human health [10].

Regarding trends in the food color industry, the use of natural pigments has increased in foods and beverages as replacements for synthetic colorants, mainly due to the health benefits of natural compounds as compared with synthetic ones [8,11]. The color change of anthocyanins, as a function of pH variation, can be an important strategy to monitor the quality of foods, indicating their shelf life in food packaging applications [12]. Others coloring agents, such as yarrow essential oil, have been used for pH-sensing in current intelligent packaging systems [13].

The search for new sources of natural colorants, as well as for knowledge concerning the stability of anthocyanins, is of great relevance, as it can vary depending on the plant matrix. As far as our knowledge is concerned, no information regarding the thermal and light stability of anthocyanin extracts of grumixama fruits is available in the literature. Therefore, for the first time, anthocyanin-rich extracts of grumixama were submitted to temperatures ranging from 30 to 100 °C, and exposed to different light irradiation, namely fluorescent, incandescent and ultraviolet, to investigate the stability kinetics of anthocyanins, with a view to future applications of these anthocyanins in food processing and storage. Data obtained herein may contribute to the definition of practical conditions that would ensure the stability of the studied anthocyanins. Consequently, anthocyanin extracts of grumixama could be properly handled, which might help in maintaining their technological and functional properties.

## 2. Materials and Methods

### 2.1. Plant Material

The full ripe (red-colored peel) and morphologically perfect grumixama fruits (*Eugenia brasiliensis* Lam.) (5 kg) were manually collected in Salvaterra, Marajó, Pará, Brazil (0° 45′ 32″ S and 48° 30′ 44″ W). The selected fruits were stored in an isothermic container and transported to the laboratory where they were washed with running water and sanitized in a sodium hypochlorite solution (100 mg/L) for 10 min, followed by rinsing with water to remove excess chlorine. These fruits were placed into a vacuum pack and stored at −18 °C until use.

### 2.2. Proximate Composition, Physicochemical Properties, and Oxidase Enzymes Activities

The grumixama fruits were analyzed by means of moisture and total solids (method n° 920,151; 105 °C), total protein (n° 920.152; nitrogen–protein conversion factor of 5.75), total lipids extracted with petroleum ether (n° 920.39), total ash (n° 940.26), pH (n° 981.12), total soluble solids (TSS; n° 932.12; °Brix), and total titratable acidity (TTA; n° 942.15; g citric acid/100 g fruit), according to the Association of Official Analytical Chemist [14]. The total carbohydrates were calculated by the following difference: [100% − (% moisture + % lipids + % proteins + % ash)]. The total energetic value was calculated according to the general Atwater conversion factors: total energetic value (kcal/100 g) = (4 kcal/g × % protein) + (4 kcal/g × % carbohydrates) + (9 kcal/g × % lipids) [15].

Polyphenoloxidase (POP) and peroxidase (POD) activities were determined by spectrophotometry at 415 nm [16] and 435 nm [17], respectively. The unit of enzyme activity (U) was defined as an increase of 0.001 absorbance units per min. Thus, the results were expressed as U/g.

### 2.3. Total Monomeric Anthocyanins, Total Phenolic Compounds and Antioxidant Capacity

Approximately 0.1 g of sample (pulp + peel) and 50 mL of 95% ethanol: HCl 1.5 M (85:15, *v*/*v*) were homogenized for 90 s. The system was filtered on Whatman n° 1 filter and the residue was washed repeatedly with acidified ethanol until it became colorless [18]. The anthocyanin-rich extract (pH 1.5) was subjected to thermal and light stability assays. Ethanol was used as a green solvent in the extraction process to allow for its application in the food industry.

Total monomeric anthocyanins (TMAs) were determined by spectrophotometry according to the pH differential method described by Wrolstad et al. [19]. An aliquot of 1 mL of anthocyanin-rich extract was homogenized with 1 mL of buffer pH 1.0 and 4.5, and the extract absorbance was performed at 520 and 700 nm after 60 min. The TMA contents were expressed as mg of cyanidin 3-glucoside equivalent (CGE)/100 g of sample.

Total phenolic compounds (TPCs) were also determined by spectrophotometry at 765 nm, according to the methodology proposed by Singleton and Rossi [20]. Briefly, 250 µL of anthocyanin-rich extract were added to 1.2 mL of Follin–Ciocalteu reagent (10%), and, after 2 min, 1 mL of sodium carbonate (7.5%) was added to the mixture and kept for 30 min protected from light. Gallic acid was used to build analytical curves ranging from 5 to 80 mg/L, and the TPC was expressed as mg of gallic acid equivalent (GAE)/100 g of sample.

The antioxidant capacity was determined by the ABTS (2,2′-azino-bis(3-ethylbenzothiazoline-6-sulfonic acid)) radical method, described by Re et al. [21]. For the reaction, 0.70 µL of anthocyanin-rich extract was added to 3 mL of ABTS reagent, and the reaction was protected from light for 10 min and read in a spectrophotometer at 734 nm. The results were expressed as mM of Trolox equivalent (TE)/100 g of sample.

### 2.4. Thermal and Light Stability of Anthocyanin Extracts

The temperature/time and light irradiation/time binomial chosen for the stability assays of anthocyanins were set according to a preliminary study [18]. The light irradiation was set using the most used light sources: fluorescent (10 W and 850 Lux), incandescent (50 W and 950 Lux) and ultraviolet. TMA and TPC were the properties used to monitor degradation of the anthocyanin extract. The thermal and light irradiation experiments were conducted until the anthocyanin concentration reached a degradation level of about 50%.

For the thermal stability experiments, the anthocyanin-rich extracts (according to item 2.3) were placed in test tubes (glass tubes) closed with lids, and covered with aluminum foil to block light incidence. The thermal stability assays were performed at the following temperatures: 30 °C, 60 °C, 80 °C and 100 °C (±2 °C). Samples were collected at time 0 for all the experiments and each 24 h for testing at 30 °C; each 1 h for testing at 60 °C; three times every 20 min and then each 1 h for testing at 80 °C; and each 10 min in the first 30 min and then each 1 h for testing at 100 °C.

**Figure 1:**
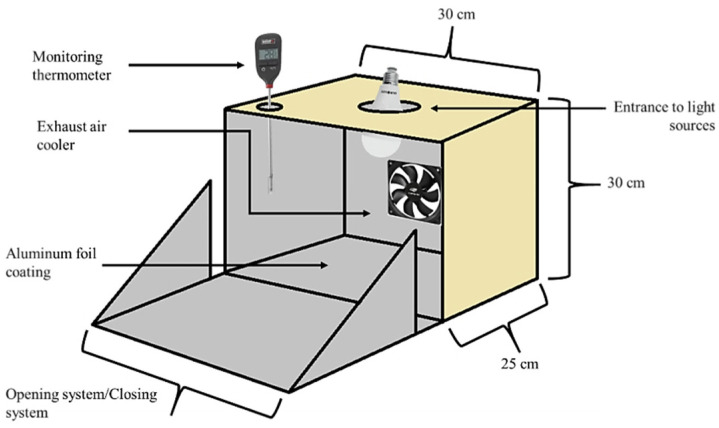
Scheme of the light irradiation box used to the light stability tests.

For the light stability experiments, the anthocyanin extracts were placed in test tubes and transferred to a light irradiation box (30 × 30 × 25 cm) with air exhausting (Figure 1). The degradation chamber with the different lights was developed to this purpose. For the experiments with fluorescent and incandescent lights, samples were taken at time 0 and every 24 h, and for ultraviolet light, the samples were taken at time 0 and every 30 min.

### 2.5. Degradation Kinetics of Anthocyanins

The thermal and light stability data were submitted to mathematical fitting using order zero (Equation (1)), first order (Equation (2)), second order (Equation (3)) and Weibull (Equation (4)) models. The half-life time (t_1/2_) for anthocyanin degradation was estimated using the Equations (5)–(8).

C = C_0_ − kt (1)

C = C_0_ exp^(−kt)^ (2)

1/C = 1/C_0_ + (kt) (3)

C = C_0_ exp[*−*kt^n^] (4)

t_1/2_ = C_0_/(2k) (for zero order reaction) (5)

t_1/2_ = −ln (0.5)/k (for first order reaction) (6)

t_1/2_ = 1/(kC_0_) (for second order reaction) (7)

t_1/2_ = ln (2)/k (for Weibull reaction) (8)

where, C_0_ and C are anthocyanin contents at time zero and time t, respectively; k is degradation rate constant; t is time; n is the form factor; and t_1/2_ is the half-life time.

The effect of temperature on degradation of anthocyanins was accessed by an Arrhenius-like equation (Equation (9)). The activation energy (E_a_) value for the process was calculated from the angular coefficient of the linear regression of k versus 1/T. The temperature quotient value (Q_10_) for the degradation of anthocyanins was calculated using Equation (10) [18].

k = k_0_e^−Ea/RT^ (9)

(10) $${\;Q}_{10}{=(k}_{2}{/k}_{1}{)}^{10/\left(T_{2}\;{-\;T}_{1}\right)}$$

where, k_0_ is the pre-exponential factor, E_a_ is the activation energy for the process degradation (kJ/mol), R is the gas constant (J/mol.K) and T is the process temperature (K).

### 2.6. Statistical Analysis

Analytical procedures were performed at least three times and the data were presented as mean and standard deviation. Data obtained with degradation studies were submitted to one-way ANOVA, followed by Tukey’s test (*p ≤* 0.05).

Order zero (Equation (1)), first order (Equation (2)), second order (Equation (3)) and Weibull (Equation (4)) models were fitted by nonlinear estimation to thermal stability data using the Statistica 7.0 program. Least squares were used to estimate the model parameters, considering the Levenberg–Marquardt algorithm, with a convergence criterion of 10^−6^. E_a_ value, estimated by regression linear.

Quality of mathematical models was evaluated by the coefficient of determination (R^2^), relative mean deviation (P) (Equation (11)) and Root Mean Square Error (RMSE) (Equation (12)).

(11) $$P=\frac{100}{N}\overset{N}{\underset{i=1}{\sum }}\frac{\left|Y_{\exp,i}{-Y}_{\mathrm{pre},i}\right|}{Y_{\exp,i}}$$

(12) $$\mathrm{RMSE}={\left[\frac{1}{N}\overset{N}{\underset{i=1}{\sum }}{\left(Y_{\exp,i\;}{-\;Y}_{\mathrm{pre},i}\right)}^{2}\right]}^{\frac{1}{2}}$$

where Y_pre_ is the predicted value, Y_exp_ is the experimental value, and N is the number of experimental measurements.

## 3. Results and Discussion

### 3.1. Characterization of Grumixama Fruits

Table 1 shows the characterization data of grumixama fruits. The moisture content of grumixama fruit (75.15%) was lower than those obtained by Teixeira et al. [22] (83.67%) for fruits of the same species from São Paulo State (Brazil). Thereafter, the studied fruits showed higher total solids content (24.85%). This result indicated that the grumixama fruits produced in Northern Brazil might be of greater interest to the food industry, as they presented higher pulp yield.

The total ash content was 4.83% dw, superior to the value observed by Ramos et al. [23] (4.08% dw) in grumixama pulp, and the total protein content (3.90% dw) was similar to that found by Teixeira et al. [22] in grumixama pulp (4.04% dw). The total lipids content was 0.28% dw, which was low when compared with another previous report (1.68% dw) [23]. The contents of sugars in fruits mainly influence their sensory characteristics and may be related to the ripening time of fruits. In this study, 38.03% dw of total carbohydrates (mainly sugars—15.16 °Brix) was found in the grumixama fruit. The total energetic value (42.31 kcal/100 g) was higher than those observed by Teixeira et al. [22] (14.24 kcal/100 g) and Silva et al. [24] (30.6 kcal/100 g). The pH value of grumixama fruit was 3.83, similar to the value reported by Ramos et al. [23] (3.23), and the total acidity was 0.31 g citric acid/100 g. The TSS/TTA ratio usually indicates a relation between acid and sour tastes. Therefore, in this study, the ratio value of 48.90 indicated a predominance of the sweeter flavor in grumixama fruits [25].

POD and PPO, along with lipoxygenase and catalase, are considered the main enzymes responsible for quality decrease in most fruits and vegetables [26]. The enzymatic activity of POP and POD were 1.34 U/g and 9.54 U/g of fruit, respectively. These enzymatic activities herein reported were much lower than those observed by Kim et al. [27], who reported 115.73 and 271.35 U/g for PPO and POD activities in blueberry fruits, respectively. The enzymatic activities of POP and POD should always be verified in food processing and storage studies as these enzymes can play a key role in the degradation of anthocyanins in the presence of cofactors and during oxidation of ascorbic acid and quinones in aerobic conditions [28,29].

The content of TMA was 2590.99 mg CGE/100 g dw in grumixama fruit. Araújo et al. [30] reported similar anthocyanin contents (2599.57 mg CGE/100 g dw) for grumixama fruits, but, for comparison purposes, the same study also showed the anthocyanin contents for other Eugenia species, such as uvaia (*E. pyriformis*) (12.02 mg CGE/100 g dw) and pitanga (*E. uniflora*) fruits (5004.44 mg CGE/100 g dw). Flores et al. [31] identified nine phenolic compounds in *E. brasiliensis* extracts, the major compounds being delphinidin 3-glucoside and cyanidin 3-galactoside: 12.2% and 76.5% of TMA, respectively, followed by cyanidin (6.1%) and delphinidin (3.4%). Other minor anthocyanins were identified with percentages less than 1.5%. Teixeira et al. [22] and Silva et al. [24] identified cyanidin 3-glucoside in grumixama pulps, accounting for 94% of total anthocyanin contents, followed by delphinidin 3-glucoside (3.5%).

The content of TPC and the antioxidant capacity were 2340.36 mg GAE/100 g dw and 728 µmol TE/100 g in grumixama fruit, respectively. Araújo et al. [30] reported lower values of TPC for araçá-boi (*Eugenia stipitata*) (782.74 mg GAE/100 g dw) and grumixama (75.08 mg GAE/100 g dw). In turn, regarding the antioxidant capacity, the same authors observed values of 99.5 µmol TE/100 g for grumixama fruit and 77.7 µmol TE/100 g for uvaia fruit, which were much lower than those found in our study.

### 3.2. Thermal Stability of Total Anthocyanins and Total Phenolic Compounds

Table 2 shows the behavior of TMA and TPC of the extracts submitted to different temperatures. The results showed that temperature increase affected the stability of TMA and TPC. Knowledge about kinetic degradation, including reaction order, rate constant and half-life time, is very important to predict loss of food quality during storage and thermal process treatments [32].

Anthocyanins showed the highest thermal stability at 30 °C, in which the TMA decreased by 52.8% after 168 h. In contrast, the exposure of anthocyanins to temperature >30 °C resulted in their degradation by 50% after 10 h at 60 °C, 1.9 h at 80 °C and 0.96 h at 100 °C. High rates of degradation could be linked to the temperature increase, which could cause breaks in the glycoside linkages of sugars in anthocyanins resulting in their derived aglycones. Aglycone moieties are more susceptible to the effect of high temperature, and, as a consequence, pigment degradation occurs faster [33]. Das et al. [34] reported that during heating of an aqueous solution of anthocyanins of purple rice bran, cyanidin 3-glucoside underwent hydrolysis, producing cyanidin aglycone by releasing a glucose molecule. According to Ursu et al. [35], thermal degradation of anthocyanins was initiated with the opening of the central ring, followed by hydrolysis of the molecule, resulting in colorless products. In turn, Hou et al. [36] reported that the stability of anthocyanins might increase via intermolecular interactions (copigmentation and self-association) when high contents of anthocyanins and other compounds (other flavonoids, for example) were observed in the extracts.

Liu et al. [37] observed that anthocyanin extracts of blueberry (pH 3.0), subjected to 60 °C, quickly degraded by 50% after 5 h of exposure and only 18.54% of the anthocyanin contents remained in the extract after 5 h of heating at 80 °C. Similar behavior was observed in the present study, indicating low retention of anthocyanins as temperature increased. Liu et al. [37] also evaluated lower temperatures (50, 40 and 25 °C) and they found that at these temperatures retention of up to 95% of the anthocyanin contents after 10 h of heating was possible. Interestingly, even following the degradation behavior with increasing temperature, the anthocyanins of grumixama extracts were more stable than the anthocyanin extracts studied by the aforementioned authors; especially at temperatures below 50 °C. This behavior may be associated to the low pH of the anthocyanin-rich extract of grumixama (pH 1.5). Liu et al. [36] observed that at low temperatures (50, 40 and 25 °C), anthocyanins showed great stability at lower pH values than at higher pH values. Therefore, a decrease in the pH of anthocyanin-rich extract of grumixama may increase the stability of anthocyanins, as low pH values keep the chemical structure of anthocyanins as that of flavylium cations, due to a proton-transfer reaction, which increases the stability of anthocyanins [34].

Peng et al. [38] evaluated the thermal stability of anthocyanin extracts from black peanut skin at temperatures above 60 °C and pH 2.5, and they reported high rates of pigment degradation. Although the behavior we observed was similar, when compared to the mentioned studies, the anthocyanins of grumixama fruits, in which the extract was at pH 1.5, were more susceptible to thermal degradation when exposed to high temperatures to that of the plant material mentioned, even at a stable pH for its chemical structure, with a range similar to that of the mentioned studies.

According to Liu et al. [37], the highest effect of temperature on the degradation of anthocyanin was associated with an increased activity of POD, which, in this study, showed higher activity than the PPO. When exposed to high temperatures and prolonged heating times, anthocyanins can undergo oxidation and structural breakdown characterized by successive reactions of deglucosylation, nucleophilic attack of water, cleavage, and polymerization [8]. These structural changes result in the loss of sugar moieties, color fading, and loss of stability, which promote a high decrease of anthocyanin contents in the exposed product [39].

Data from Table 2 were mathematically fitted to zero order, first order, second order and Weibull models. Mathematical modeling for the thermal degradation kinetics of pigments, such as anthocyanins, can be used to predict residual degradation after heat treatment and to elucidate degradation mechanisms. The statistical parameters of R^2^, RMSE and P were used to evaluate the quality of the fits. The values of the parameters found for the fit to the zero-order model (R^2^ > 0.780, RMSE < 0.102 and P < 5.78%), first-order model (R^2^ > 0.897, RMSE < 0.070 and P < 0.80%), second order model (R^2^ > 0.951, RMSE < 0.047 and P < 1.75%) and Weibull model (R^2^ > 0.963, RMSE < 0.038 and P < 1.34%) presented a good fit of these models to all the experimental data for the degradation of the anthocyanins.

**Figure 2:**
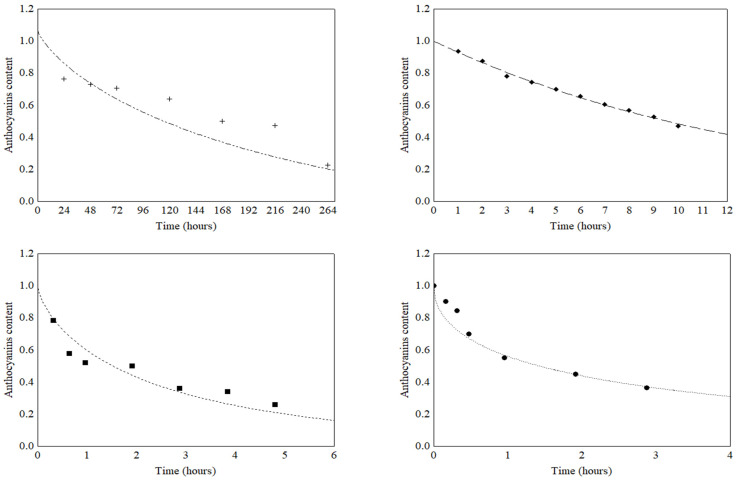
Weibull model fitted to the thermal degradation data of anthocyanins in grumixama extracts. (+) 30 °C; (◆) 60 °C; (■) 80 °C; (●) 100 °C.

However, the highest R^2^ values found in combination with the lowest RMSE and P values suggested that the Weibull model was the one that had the best fit for the experimental data for anthocyanin degradation and presented predictive capacity. In addition, this model is highly recommended due to its mathematical simplicity (number of parameters) and its great flexibility, which favors its use for practical purposes. The thermal degradation curves of anthocyanins in the grumixama extract, obtained by the Weibull model for the different temperatures, are shown in Figure 2.

The parameters for the mathematical modeling of the Weibull model (k, n and R^2^) and the kinetic parameters (Q_10_, t_1/2_ and E_a_) obtained by the model are shown in Table 3. The parameter k; reaction rate constant, or scale factor, is related to the rate of degradation of anthocyanins. The higher the value of k, the faster was the degradation of the pigment, which was observed for the degradation of anthocyanins in the grumixama extract as temperature increased. The thermal degradation curves of anthocyanins at 30 °C, 80 °C and 100 °C showed the same trend (Figure 2), with the concavity facing upwards, which was confirmed by the reaction order (*n* < 1) (Table 3). This behavior suggested that, for the anthocyanin-rich extract of grumixama, there was a labile fraction and a heat resistant fraction. In turn, the degradation curve at 60 °C presented a value equal to 1, which was a particular case in which the Weibull model was equal to the first-order kinetics. In this case, the first-order model could also be used to predict the degradation behavior of anthocyanins in the grumixama extract. However, the Weibull model is more suitable because it takes into account the variability of n over time and has great flexibility, which favors its use for practical purposes.

Thermal degradation of anthocyanins can result in a variety of degradation products and intermediate compounds, depending on the conditions of heating and the processing time. The mechanisms of anthocyanin degradation are not fully elucidated, but it is known that the degradation rates of this pigment increase during processing and storage, as temperature increases [32]. According to Das et al. [34], during thermal treatments, the chemical structures of cyanidin 3-glucoside and peonidin 3-glucoside may undergo modifications, depending on the severity and nature of heating. These authors observed that cyanidin 3-glucoside showed higher stability during thermal treatments.

The t_1/2_ is the required time to decrease the concentration of the monitored compounds by half of the initial value. Data showed that the increase in temperature favored a decrease in time for the degradation of anthocyanin (Table 3). Peng et al. [38] reported t_1/2_ of 73.51 h, 31.97 h, 18.10 h and 11.05 h for thermal degradation of black peanut skin anthocyanins, in the temperature range from 60 °C to 90 °C, as well as an increase in constant k values with temperature increase. Liu et al. [37] observed k values of 0.008, 0.026 and 0.146 h^−1^ for temperatures at 25, 60 and 80 °C, and t_1/2_ values of 91.06, 26.51 and 4.73 h for the same temperatures, respectively. In turn, Bolea et al. [40] reported k values varying from 10.46 h^−1^ (60 °C) to 12.25 h^−1^ (100 °C) for thermal degradation of black rice anthocyanins. These values were much higher than those observed in our study for the same temperatures, which corroborated the greater thermal stability of grumixama anthocyanins.

The factor Q_10_ is the reaction acceleration factor as a function of temperature and expresses, therefore, how much the rate of a given change depends on temperature. Higher values of Q_10_ indicate greater influence of temperature on degradation processes or greater acceleration of processes with temperature increase. The highest Q_10_ value was observed in the 60–80 °C range (2.82), which indicated that, within this range, the degradation of anthocyanins was strongly affected by the temperature process.

The energy E_a_ is the minimum energy required to start a given reaction, indicating its sensitivity to temperature. Therefore, E_a_ is a valuable parameter to characterize temperature dependence of anthocyanin degradation. A high E_a_ value was found (52.67 kJ/mol), which meant high susceptibility of the anthocyanins of grumixama when exposed to high temperatures. However, the anthocyanins of grumixama were less susceptible to degradation when compared to the study by Martynenko and Chen [41], which obtained zero order kinetics, a value of E_a_ of 61.98 kJ/mol, and a Q_10_ value of 1.85 for the temperature range of 70 to 80 °C for anthocyanin degradation of the blueberry in the hydrothermodynamic process. In studies on the anthocyanin degradation of juçara and “italia” grapes, Peron, Fraga and Antelo [42] observed first order kinetics with high values of Q_10_ (4.4 and 3.5, respectively) for a temperature range of 50 to 70 °C.

The mathematic models were not fitted to the TPC data, because the TPC degradation presented different behaviors as a function of temperature. It was possible to observe better behavior for TPC degradation at 30 °C and 60 °C, requiring 168 h to reduce the TPC content to 63% at 30 °C and 9 h to reduce the TPC content to 51% at 60 °C (Table 2). At high temperatures, the degradation of anthocyanins can generate other compounds that react with the Folin–Ciocalteu reagent, resulting in overestimation of the results [43], which could justify the oscillation observed on regard to TPC. Tsali and Goula [44] reported an increase in phenolic compounds retention in encapsulated grape pomace during storage, which was partially explained by the hydrolysis of conjugated polyphenols.

### 3.3. Light Stability of Total Anthocyanins and Total Phenolic Compounds

Table 4 shows the values obtained for the degradation kinetics of TMA and TPC in the grumixama extracts exposed to fluorescent, incandescent and ultraviolet light. All types of light studied exhibited a significant effect on the stability of the anthocyanins of grumixama. Although the stability of anthocyanins observed for the fluorescent and incandescent light exposures showed similar behaviors, the degradation rate was higher under fluorescent incidence (k = 0.015 h^−1^) (Table 5). Both required 120 h to decrease the anthocyanin content by 50%. On the other hand, under ultraviolet irradiation, the stability of the anthocyanins was highly affected (k = 3.748 h^−1^), as it took less than 10 min for the anthocyanin content to be reduced by half. Additionally, the stability of anthocyanins was superior under fluorescent and incandescent lights when compared to most of the thermal treatments (60–100°C) (Table 2), while under ultraviolet radiation, the degradation rate of anthocyanins was extremely high. Photons of UV light are absorbed by organic molecules and affect conjugated double bonds, such as in aromatic rings, double rings and compounds including disulfide bonds [45], and, therefore, may reduce the stability of anthocyanins.

**Figure 3:**
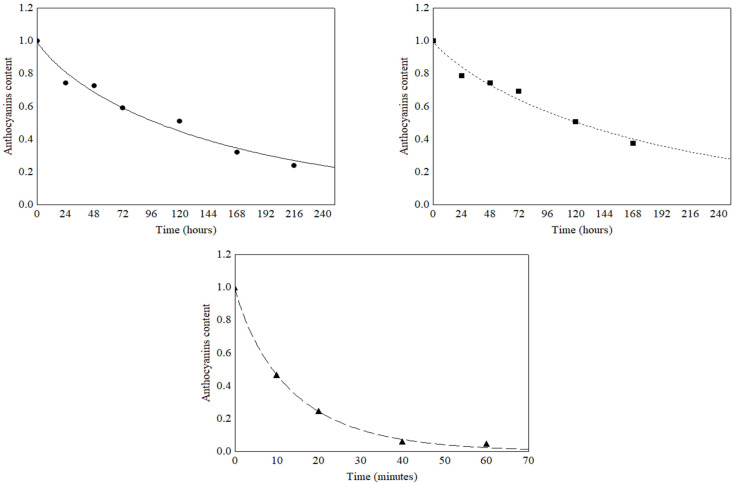
Weibull model fitted to the light degradation data of anthocyanins in grumixama extracts. (●) Fluorescent, (■) Incandescent and (▲) Ultraviolet lights.

The data on the degradation of anthocyanins, due to light exposure, (Table 4) were subjected to mathematical modeling and the quality of the fits was evaluated by the parameters R^2^, RMSE and P (Table 5). The results found for the zero-order (R^2^ > 0.752; RMSE < 0.134 and P < 9.56%), first-order (R^2^ > 0.973; RMSE < 0.034 and P < 5.88%), second order (R^2^ > 0.965; RMSE < 0.047 and P < 18.13%) and the Weibull model (R^2^ > 0.981; RMSE < 0.028 and P < 2.48%) models indicated that the Weibull model presented the best fit to predict the degradation of anthocyanins submitted to fluorescent, incandescent and ultraviolet light. The degradation curves obtained by the Weibull model are shown in Figure 3. The parameters for the mathematical modeling of the models (k, n and t_1/2_) and the kinetic parameter t_1/2_ obtained by the models are shown in Table 5.

The observed data showed that the type of light had a marked effect on the k and t_1/2_ values for the anthocyanin’s degradation. The lower value of k observed for the anthocyanins after incandescent light exposure promoted a higher t_1/2_ value (59.63 h) when compared to the t_1/2_ value of 45.61 h obtained by the fluorescent light. In turn, the ultraviolet light showed the highest k values, and, consequently, the smallest t_1/2_ value (0.18 h).

The kinetic orders for the anthocyanin degradation of grumixama extracts also differed to those reported in the literature, which normally indicate that anthocyanin degradation follows the zero or first-order kinetics. Chisté et al. [18] investigated the stability of anthocyanin extracts from mangosteen peel under different light irradiations and reported first-rate degradation kinetics and high t_1/2_ values, being 597 h, 306 h, 177 h and 100 h for fluorescent, incandescent, ultraviolet and infrared lights, respectively. The anthocyanins of grumixama, compared to those of the aforementioned authors, were more susceptible to degradation, according to the lower t_1/2_ values. The differences observed could be explained by the structural configuration of anthocyanins in different plant matrices, and also the presence of other compounds by intra- and intermolecular stacking, such as acylation, copigmentation and self-association, that may protect anthocyanins from degradation factors [46]. According to the literature, the main anthocyanins found in mangosteen peel are cyanidin 3-sophoroside and cyanidin 3-glucoside, while cyanidin 3-glucoside is the majority anthocyanin in grumixama [47]. Delgado-Vargas et al. [47] explained that the degree of glycosylation was also responsible for increasing the stability of these molecules (di-glycosylated anthocyanins were more stable than the monoglycosylated anthocyanins).

The light degradation data for TPC (Table 4) were not modeled mathematically, because the results showed irregular behavior, without a defined trend, as observed for the thermal treatments. The fluorescent and incandescent light took 120 min to reduce the TPC content by 62% and 37%, respectively. Del-Toro-Sánchez et al. [48] studied the stability of extracts from *Anemopsis californica* at 25 and 50 °C, combined with exposure to light, and reported higher values for TPC than the values observed for light degradation of TPC in grumixama extracts. According to Zhang et al. [49], among all phenolic compounds, anthocyanins are the most susceptible to degradation. In previous studies, anthocyanins were reported to decompose to phloroglucinaldehyde and benzoic acid derivatives during storage at different temperatures, which might be the reason for the variation and increase observed for TPC, since these products also react with Folin–Ciocalteu reagent, and perhaps, led to overestimation of the TPC [50,51,52].

## 4. Conclusions

The grumixama fruits showed high contents of TMA and TPC, and promising antioxidant capacity. The results showed that the temperature range from 60 to 80 °C was critical to the stability of grumixama anthocyanins, and that temperatures below 50 °C could effectively preserve these anthocyanins. Incandescent and fluorescent lights weakly affected the anthocyanin stability, while anthocyanins were quickly degraded by UV light. Therefore, anthocyanin extracts from grumixama fruits should be handled at temperatures lower than 50 °C and protected from UV light exposure. Further studies are needed to evaluate the stability of the anthocyanins of grumixama fruits under different food processing conditions, such as pasteurization, or even studies concerning the gastrointestinal digestion of grumixama fruits.

## Figures and Tables

**Table 1:** Proximate composition, physicochemical properties and enzyme activity of grumixama fruit (*Eugenia brasiliensis* Lam).

Property/Component	Grumixama Fruit
Proximate composition	
Moisture (g/100 g fruit)	75.15 ± 0.88
Total solids (TS) (g/100 g fruit)	24.85 ± 0.88
Ash (g/100 g dw)	4.83 ± 0.08
Total proteins (g/100 g dw)	3.90 ± 0.20
Total lipids (g/100 g dw)	0.28 ± 0.04
Total carbohydrates (g/100 g dw)	38.03 ± 0.16
Total energetic value (kcal/100 g fruit)	42.31 ± 1.04
Physicochemical properties	
pH	3.83 ± 0.05
TTA (g citric acid/100 g fruit)	0.31 ± 0.01
TSS (°Brix)	15.16 ± 0.08
TSS/TTA ratio	48.90 ± 0.10
Enzyme activity	
PPO activity (U/g fruit)	1.34 ± 0.05
POD activity (U/g fruit)	9.54 ± 0.14
Bioactive compounds	
TMA (mg CGE/100 g dw)	2590.99 ± 40.32
TPC (mg GAE/100 g dw)	2340.36 ± 47.48
Antioxidant capacity (µmol TE/100 g fruit)	728.00 ± 35.69

dw—dry weight; TTA—Total titratable acidity; TSS—Total soluble solids; PPO—Polyphenol oxidase; POD—Peroxidase; TMA—Total monomeric anthocyanins; TPC—Total phenolic compounds; CGE—Cyanidin 3-glucoside equivalent; GAE—Gallic acid equivalent; TE—Trolox equivalent.

**Table 2:** Normalized values of TMA and TPC degradation by thermal processes.

Property	Temperature	Time (h)										
		0	24	48	72	120	168	216	264			
TMA	30 °C	1.00 ± <0.01 ^a^	0.76 ± 0.04 ^b^	0.72 ± 0.07 ^b^	0.70 ± <0.01 ^b^	0.63 ± 0.04 ^b^	0.50 ± 0.03 ^c^	0.47 ± <0.01 ^c^	0.22 ± <0.01 ^d^			
TPC		1.00 ± 0.09 ^a^	0.87 ± 0.08 ^ab^	0.80 ± <0.01 ^abc^	0.72 ± 0.04 ^bcd^	0.56 ± 0.01 ^de^	0.55 ± 0.03 ^cde^	0.54 ± <0.01 ^de^	0.37 ± 0.03 ^e^			
		**0**	**1**	**2**	**3**	**4**	**5**	**6**	**7**	**8**	**9**	**10**
TMA	60 °C	1.00 ± <0.01 ^a^	0.93 ± 0.03 ^ab^	0.87 ± 0.04 ^abc^	0.77 ± 0.01 ^bcd^	0.74 ± <0.01 ^d^	0.69 ± <0.01 ^d^	0.65 ± ≤0.01 ^def^	0.60 ± ≤0.01 ^def^	0.56 ± 0.03 ^ef^	0.52 ± 0.12 ^ef^	0.46 ± 0.01 ^d^
TPC		1.00 ± 0.08 ^a^	0.92 ± 0.04 ^a^	0.90 ± 0.05 ^a^	0.85 ± 0.07 ^ab^	0.76 ± 0.03 ^abc^	0.77 ± 0.03 ^abc^	0.74 ± 0.01 ^abc^	0.63 ± 0.01 ^bc^	0.53 ± ≤0.01 ^c^	0.49 ± <0.01 ^d^	
		**0**	**0.32**	**0.64**	**0.96**	**1.92**	**2.88**	**3.84**	**4.80**			
TMA	80 °C	1.00 ± 0.02 ^a^	0.78 ± 0.08 ^b^	0.57 ± 0.03 ^c^	0.57 ± 0.03 ^cd^	0.49 ± 0.01 ^cde^	0.36 ± 0.02 ^cde^	0.33 ± 0.02 ^de^	0.26 ± <0.01 ^e^			
TPC		1.00 ± 0.06 ^a^	0.92 ± 0.01 ^a^	0.71 ± 0.04 ^b^	0.71 ± 0.04 ^ab^	0.85 ± 0.03 ^ab^	0.77 ± 0.02 ^ab^	0.81 ± <0.01 ^ab^	0.72 ± <0.01 ^b^			
		**0**	**0.16**	**0.32**	**0.48**	**0.96**	**1.92**	**2.88**	**3.84**			
TMA	100 °C	1.00 ± 0.03 ^a^	0.90 ± 0.01 ^a^	0.84 ± 0.09 ^ab^	0.69 ± 0.06 ^bc^	0.54 ± 0.03 ^cd^	0.44 ± <0.01 ^de^	0.36 ± 0.01 ^e^	0.23 ± 0.01 ^f^			
TPC		1.00 ± 0.06 ^ab^	0.97 ± 0.10 ^ab^	0.98 ± 0.04 ^ab^	1.03 ± 0.03 ^ab^	1.08 ± 0.07 ^ab^	1.21 ± 0.05 ^a^	0.87 ± 0.02 ^b^	0.97 ± 0.02 ^b^			

Values measured until TMA and TPC decreased by 50% from the initial value. TMA—Total monomeric anthocyanins; TPC—Total phenolic compounds. Means (±standard deviations) with different superscript letters in the same row are statistically different (*p* ≤ 0.05, Tukey test).

**Table 3:** Parameters of mathematical modeling and kinetic parameters of thermal degradation of anthocyanins in grumixama extracts obtained by the Weibull model.

T (°C)	k (h^−1^)	n	R^2^	Q_10_	t_1/2_ (h)	E_a_ (kJ/mol)
30	0.015	0.76	0.90	1.70 (30–60 °C)	46.82	52.67 (R^2^ = 0.92)
60	0.073	1.00	0.99	2.82 (60–80 °C)	9.50	
80	0.580	0.50	0.97	0.94 (80–100 °C)	1.19	
100	0.517	0.70	0.97		1.34	

T—temperature (°C), Q_10_—degradation quotient (dimensionless), k—reaction constant (h^−1^), n—reaction order, t_1/2_—half-life time (h), E_a_—activation energy (kJ/mol) and R^2^—determination coefficient.

**Table 4:** Normalized values of TMA and TPC degradation by light exposure.

Response	Light	Time (h)						
		0	24	48	72	120	168	216
TMA	Fluorescent	1.00 ± 0.09 ^a^	0.74 ± 0.09 ^ab^	0.72 ± 0.11 ^ab^	0.59 ± 0.01 ^b^	0.50 ± 0.01 ^bc^	0.32 ± 0.04 ^c^	0.23 < 0.01 ^c^
TPC		1.00 ± 0.09 ^a^	0.73 ± 0.06 ^ab^	0.67 ± 0.01 ^abc^	0.62 ± 0.02 ^bc^	0.38 ± 0.05 ^d^	0.49 ± 0.06 ^cd^	0.48 ± 0.06 ^cd^
		**0**	**24**	**48**	**72**	**120**	**168**	**216**
TMA	Incandes-cent	1.00 ± 0.05 ^a^	0.78 ± 0.02 ^ab^	0.74 ± 0.01 ^bc^	0.69 ± 0.01 ^bc^	0.50 ± 0.01 ^cd^	0.37 ± 0.02 ^de^	
TPC		1.00 ± 0.01 ^a^	0.71 ± 0.04 ^bc^	0.57 ± <0.01 ^c^	0.47 ± 0.02 ^c^	0.63 ± <0.01 ^bc^	1.11 ± 0.22 ^ab^	0.92 ± 0.01 ^ab^
		**0**	**10**	**20**	**40**	**60**		
TMA	Ultraviolet	1.00 ± 0.27 ^a^	0.46 ± 0.06 ^b^	0.24 ± 0.02 ^bc^	0.05 ± 0.01 ^bc^	0.04 ± <0.01 ^bc^		
TPC		1.00 ± 0.15 ^a^	0.60 ± <0.01 ^ab^	0.88 ± 0.04 ^ab^	0.60 ± 0.04 ^ab^	0.53 ± 0.07 ^b^		

Values measured until TMA and TPC decreased by 50% from the initial value. TMA—Total monomeric anthocyanins; TPC—Total phenolic compounds. Means (±standard deviations) with different superscript letters in the same row are statistically different (*p* ≤ 0.05, Tukey test).

**Table 5:** Parameters of mathematical modeling and kinetic parameters of light degradation of anthocyanins in grumixama extracts obtained by the Weibull model.

Light	k (h^−1^)	n	t_1/2_ (h)
Fluorescent	0.015	0.82	45.61
Incandescent	0.012	0.85	59.63
Ultraviolet	3.748	0.88	0.18

k—reaction constant (h^−1^), n—reaction order, t_1/2_—half-life time (h).

## Data Availability

Data is contained within the article.
